# Treatment of Malignant Gliomas in Elderly Patients: A Concise Overview of the Literature

**DOI:** 10.1155/2014/734281

**Published:** 2014-04-22

**Authors:** Patrizia Farina, Giuseppe Lombardi, Eleonora Bergo, Anna Roma, Vittorina Zagonel

**Affiliations:** Medical Oncology 1 Unit, Veneto Institute of Oncology IOV-IRCCS, 35128 Padua, Italy

## Abstract

Gliomas are the most frequent primary brain tumors and the incidence data has increased in the elderly population. Unfortunately, prospective studies on this population are few and so the right treatment is unknown. In the elderly patients no standard treatment has been established and therefore the optimal treatment should be individualized. We performed a review analyzing the prognostic and predictive factors, the clinical studies, and the correct management of this population.

## 1. Introduction

Gliomas are the most frequent primary brain tumors and the incidence data has increased in the elderly population. The elderly population is growing in many countries; therefore, the number of GBM patients diagnosed at age >65 is expected to continue to increase [1]. Clinical management of elderly patients with primary brain tumors is difficult, owing to multiple comorbidities, polypharmacy, decreased tolerance to chemotherapy, and an increased risk of radiation-induced neurotoxicity. Although survival is inferior in older GBM patients, age alone should not disqualify patients from aggressive therapy with surgical resection, radiotherapy, and chemotherapy [2]. In fact, the therapeutic approach is also influenced by other prognostic factors such as grade, Karnofsky performance status (KPS), and comorbidity. Surgery remains the first-line management strategy to obtain the histological proof of the diagnosis and to determine the tumor molecular profile. After surgery, the standard treatment of gliomas includes chemotherapy combined with or without radiotherapy after the radical resection. Supportive care and treatment with corticosteroids and antiepileptics (AEs) play an important role in elderly patients [3].

## 2. Epidemiology

In adults, glioblastoma represents 50% of gliomas, while grade III and grade II represent 25% of the other tumor cases [4]. Glioblastoma is among the most aggressive tumor types. Its prognosis is associated with a rapidly progressive disease and a generally fatal outcome. While the incidence of glioblastoma and anaplastic astrocytoma has increased in the older patients, the low-grade tumors are less frequent in the elderly population than in the younger population and pilocytic astrocytomas are extremely rare in these patients [5]. Patients aged >65 years represent a third of all glioblastoma patients, and their total number is increasing. In fact, the peak incidence is between 65 and 84 years. Moreover, elderly patients have been reported to have a 3.18-fold higher relative risk of brain tumor compared with young adults [6]. Chakrabarti et al. reported age-specific incidence rates among males aged 70–74 years and females aged 75–79 years and found that males had a 60% increased risk of brain tumors. Often in the elderly, the diagnosis of brain cancer can be more difficult because clinical features can be confusing [7]; a retrospective study of 393 elderly glioblastoma patients reported that the most common symptoms seem to be cognitive disorders and hemiparesis; headache and intracranial hypertension are also observed in some patients. Differential diagnosis must be made with cognitive deterioration due to dementia or delirium. And so, very important is the role of neuroimaging [8].

## 3. Prognostic Factors and Molecular Alterations

The phenotype (astrocytic or oligodendrocytic) and grade are strongly correlated with outcome. Age and performance status (PS) are other important clinical prognostic factors in glioma patients. Elderly patients have a worse prognosis than younger patients [2]. Therefore, the therapeutic approach in these patients has to consider the age, grade, PS, and comorbidities. Iwamoto et al. analyzed 394 patients with a diagnoses of GBM with ≥65 years: older age, KPS, and extent of tumor resection were found to be independent prognostic factors. Although survival is inferior in older GBM patients, age alone should not disqualify patients from aggressive therapy with surgical resection, RT, and chemotherapy [8]. Several molecular alterations have been identified as prognostic factors. One of the most important factors is the 1p19q codeletion, which has prognostic and predictive value in the progression of glioma. Tumors that contain this codeletion have been shown to be associated with an oligodendroglial phenotype, a slower course of the disease and a better response to chemotherapy. However, this molecular alteration has not been evaluated in elderly patients [9].

Methylation of the O^6^-methylguanine-DNA methyltransferase (MGMT) promoter has been described to be associated with a better prognostic value and potentially a better response to alkylating agents. In the study of Reifenberger et al. [10], 233 glioblastoma patients aged ≥70 years found an MGMT methylation rate of 57.5%. It seems that elderly patients with methylated MGMT tumors presented with longer progression-free survival (PFS) and overall survival (OS) and were more likely to benefit from chemotherapy. Patients with MGMT methylated tumors had longer PFS when treated with radiotherapy (RT) plus chemotherapy (CT) or CT alone compared to patients treated with RT alone. Patients with MGMT unmethylated tumors appeared to derive no survival benefit from CT, regardless of whether given at diagnosis together with RT or as a salvage treatment. In this study patients treated with RT plus CT or CT alone demonstrated longer OS when pyrosequencing revealed >25% MGMT methylated alleles.

Isocitrate dehydrogenase 1 (IDH1) mutations have been reported to be expressed in low-grade, anaplastic glioma, and secondary glioblastoma; this mutation seems to be associated with better clinical outcomes for patients. However, a recent molecular analysis performed in an elderly population revealed that only 2% of patients had the IDH1 mutation, precluding the determination of an association between IDH mutation and outcome for these patients [11]. Other molecular alterations have been reported as TP53, but their prognostic implication remains unclear.

## 4. Management

### 4.1. Surgery

Although the optimal treatment for elderly patients with GBM remains controversial, the common treatment is tumor resection or biopsy followed by radiotherapy. When possible, tumor complete resection is recommended to reduce symptoms and to increase the efficacy of adjuvant treatment. Neurosurgery in elderly patients is more risky than in younger patients. Kita et al. [12] analyzed 389 patients over 60 years with glioblastoma in a retrospective study between 1980 and 1994 and found that surgical resection versus biopsy alone decreases survival. The authors evaluated 715 glioblastoma patients diagnosed during 1980–1994 in the Canton of Zurich, Switzerland, to provide information on how patients were treated at the population level. Despite a general policy during the study period of treatment by surgical intervention aimed at maximum tumor removal followed by radiotherapy, there was a marked tendency toward limited treatment with advancing patient age. Of those younger than 65 years, 82% were treated either with surgery followed by radiotherapy, surgery alone, or radiotherapy alone versus 47% of patients 65 years or older. Only 25% of patients older than 75 years underwent surgery and/or radiotherapy, while the remaining patients were given best supportive care (BSC). The mean ages of patients were 54.5 years for those treated with surgery and radiotherapy, 58.3 years for surgery alone, 62.2 years for radiotherapy alone, and 69.2 years for BSC. Among patients who were treated with surgery plus radiotherapy and those treated with radiotherapy alone, younger patients (<60 years) had a significantly higher survival rate than older patients (> or =60 years). In contrast, no significant difference in survival was observed between younger and older patients treated with surgery alone or receiving BSC, suggesting that lower survival rates in elderly patients with glioblastoma may be at least in part due to a lesser response to radiotherapy.

In the study by Iwamoto et al. [8], three hundred ninety-four GBM patients with a median age of 71.9 years (59% of whom were men) were analyzed. Approximately 18% of patients underwent biopsy, whereas 82% underwent tumor resection; 81% received radiotherapy (RT), and 43% received adjuvant chemotherapy. The median overall survival was 8.6 months; at the time of last follow-up, 90% of patients had died, and the median follow-up of the 39 surviving patients was 12 months. In a multivariate analysis, younger age, better Karnofsky performance status (KPS), single tumor, and surgical resection were found to be independent predictors of survival. Comparing 103 patients who received adjuvant chemotherapy with 48 who were only followed after RT, there was a 55% decrease in the risk of death (hazards ratio, 0.45; 95% CI, 0.30–0.66 (*P* < 0.0001) after adjusting for age, KPS, extent of surgical resection, and number of lesions. From this study it emerged that, similar to studies on younger GBM patients, advancing age, KPS, and extent of tumor resection were found to be independent prognostic factors in the current study. Although survival is inferior in older GBM patients, age alone should not disqualify patients from aggressive therapy with surgical resection, RT, and chemotherapy.

### 4.2. Radiotherapy

Barker et al. [13] analyzed survival of elderly patients with GBM treated with chemo-radiotherapy concomitant. In this study, 291 patients with >65 years were analyzed. Concurrent TMZ and RT improved median survival of patients with favorable prognostic factors from 12 to 21 months and from 10 to 13 months in patients 65–70 and >71 years old. And so, survival of elderly patients with GBM seems to be prolonged with the use of concomitant TMZ during RT. Mohan et al. [14] analyzed elderly patients (> or =70 years) who had primary treatment for glioblastoma multiforme from 1977 to 1996. The study group (*n* = 102) included 58 patients treated with definitive radiation, 19 treated with palliative radiation, and 25 who received no radiation. To compare the results with published findings, the authors grouped the patients according to the applicable prognostic categories developed by the Radiation Therapy Oncology Group (RTOG): RTOG group IV (*n* = 6), V (*n* = 70), and VI (*n* = 26). Patients were retrospectively assigned to prognostic groups IV, V, or VI based on age, performance status, extent of surgery, mental status, neurologic function, and radiation dose. Treatment included surgical resection and radiation (*n* = 49), biopsy alone (*n* = 25), and biopsy followed by radiation (*n* = 28). Patients were also stratified according to whether they were optimally treated (gross total or subtotal resection with postoperative definitive radiation) or suboptimally treated (biopsy, biopsy + radiation, surgery alone, or surgery + palliative radiation). Patients were considered to have a favorable prognosis (*n* = 39) if they were optimally treated and had a Karnofsky performance status (KPS) score of at least 70. The median survival for patients according to RTOG groups IV, V, and VI was 9.2, 6.6, and 3.1 months, respectively. The median overall survival was 5.3 months. The definitive radiation group (*n* = 58) had a median survival of 7.3 months compared to 4.5 months in the palliative radiation group (*n* = 19) and 1.2 months in the biopsy-alone group. Optimally treated patients had a median survival of 7.4 months compared to 2.4 months in those suboptimally treated. The favorable prognosis group had an 8.4-month median survival compared to 2.4 months in the unfavorable group. On multivariate analysis, the KPS, RTOG group, favorable/unfavorable prognosis, and optimal treatment/suboptimal treatment were significant predictors of survival. From this study it emerged that elderly patients with good performance status (> or =70 KPS) when treated aggressively with maximal resection and definitive radiation had longer survival than those treated with palliative radiation and biopsy. Aggressive treatment in such patients should be considered.

The radiochemotherapy treatment with alkylating agents such as temozolomide is the standard treatment in adult glioblastoma patients. The RT schedule in elderly brain tumor patients is 60 Gy in 30 fractions of 2 Gy, although the hypofractionated schedule in 15 fractions is used in some treatment centers. Hypofractionated RT (HSRT) is an interesting alternative in elderly patients because it shortens the treatment duration. Minniti et al. [15] evaluated the efficacy of reirradiation and systemic chemotherapy as salvage treatment in patients with recurrent malignant glioma. Fifty-four patients with recurrent malignant glioma received hypofractionated stereotactic radiotherapy (HSRT) plus systemic therapy. All patients had Karnofsky performance score ≥60 and were previously treated with standard conformal RT (60 Gy) with concomitant and adjuvant temozolomide (TMZ) up to 12 cycles. Thirty-eight patients had a GBM and 16 patients had a grade 3 glioma. At the time of recurrence, all patients received HSRT (30 Gy in 6-Gy fractions) plus concomitant TMZ (75 mg/m(2)/day) followed by continuous TMZ at 50 mg/m(2) everyday up to 1 year or until progression. Median overall survival after HSRT was 12.4 months, and the 12- and 24-month survival rates were 53 and 16%, respectively. PFS was 6 months, and the 12- and 24-month PFS rates were 24 and 10%, respectively. KPS > 70 and grade 3 glioma were independent favourable prognostic factors for survival. In general chemoradiation regimen was well tolerated with relatively low treatment-related toxicity. HSRT plus concomitant TMZ followed by continuous dose-intense TMZ is a feasible treatment option associated with survival benefits and low risk of complications in selected patients with recurrent malignant glioma.

In a French study, the outcomes in elderly patients with newly diagnosed glioblastoma after fractionated radiotherapy and concomitant TMZ were analyzed; in the study, 44 patients aged over 70 years were analyzed, all the patients underwent surgery, 38 of these patients had been treated with hypofractionated RT and 6 patients with normal schedule. The concomitant chemotherapy was performed on 35 patients and adjuvant therapy in 20 patients. The median recurrence onset was 6.7 months. The study reports that hypofractionated radiotherapy and chemotherapy with temozolomide are well accepted in elderly patients [16]. The study by Villà et al. [17] found that elderly patients may benefit from hypofractionated RT, while benefit from chemotherapy can only be found in patients with MGMT methylation gene. Age above 65 years is a strong negative prognostic factor for survival in patients with malignant gliomas (MG) treated with radiotherapy (RT) and its value has been questioned. The authors analyzed the effect of RT on the survival of elderly patients with malignant gliomas. They examined 85 consecutive elderly patients with a histological diagnosis of MG. Age ranged from 65 to 81 years (median 70 years). Glioblastoma multiforme (GBM) was diagnosed in 64 patients (75.3%). Surgical treatment included needle biopsy in 32 patients (37.6%). Median postoperative Karnofsky performance status (KPS) was 60 (range: 30–100). Survival probability was estimated using Kaplan-Meier method and compared with the log-rank test. Median survival time for all patients was 18.1 weeks. In multivariate analysis, RT was the only independent prognostic variable for survival (HR: 9.1, 95% CI 4.5–18.7). Forty-two patients did not start RT mostly due to low KPS (<50). The median survival of the 43 patients who started RT was 45 weeks. In these patients, Cox multivariate analysis indicated that age was independently associated with prolonged survival (HR: 2.85, 95% CI 1.31–6.19). Median survival of patients aged 70 years and younger was 55 weeks compared with 34 weeks for patients older than 70 years. From this study it emerged that the overall survival for elderly patients with MG is poor. RT seems to improve survival in patients up to 70 years, but in older patients treated with RT the survival is significantly shorter.

In the study by Saito et al., the benefits and adverse events of combination therapy were analyzed. In this study, 76 patients with newly diagnosed glioblastoma, treated with standard radiotherapy and chemotherapy, were compared to two groups: the first group of 27 patients (aged over 65 years) and the second group of 49 patients (under the age of 65 years). The incidence of common side effects was higher in patients aged over 65 years and cognitive disorders were observed only in the group of elderly patients. Of the 76 newly diagnosed glioblastoma patients who were treated with standard radiotherapy (60 Gy/30 fractions) and TMZ, treatment toxicity and therapeutic outcome were evaluated in 27 elderly patients (age 65 years or older) and compared with those of 49 nonelderly counterparts (age younger than 65 years). The incidence of common toxicity criteria grade 4 adverse events during the concomitant course was higher in the elderly group than that in the nonelderly group (26% versus 8%). Cognitive dysfunction was observed only in the elderly group. The median overall survival (OS) and median progression-free survival in the elderly group were 15.2 months and 8.4 months, respectively. OS was significantly shorter in the elderly group than in the nonelderly group (*P* = 0.021). TMZ-based chemoradiotherapy was associated with an increased risk of grade 4 adverse events in the elderly patients during concomitant use. From this study it emerged that treatment of glioblastoma in elderly patients must be optimized to reduce toxicity to acceptable levels and to maintain efficacy [18].

Minniti et al. [19] conducted a prospective trial in 32 consecutive elderly patients with glioblastoma who underwent surgery followed by radiotherapy plus concomitant and adjuvant temozolomide. Standard RT plus concomitant and adjuvant temozolomide seems to be a feasible treatment for elderly patients, with newly diagnosed glioblastoma, presenting with good prognostic factors. The primary endpoint of the study was overall survival (OS). Secondary endpoints included progression-free survival (PFS) and toxicity. The median OS was 10.6 months and the median PFS was 7 months. The 6-month and 12-month survival rates were 91% and 37%, respectively. The 6-month and 12-month PFS rates were 56% and 16%, respectively. In multivariate analysis, KPS was the only significant independent predictive factor of survival (*P* = 0.01). Adverse effects were mainly represented by neurotoxicity (40%), resolved in most cases with the use of steroids, and grade 3 and grade 4 hematologic toxicity in 28% of patients. Chemotherapy was stopped in 2 patients, delayed in 9 patients, and reduced in 4 patients. From the study it emerged that standard RT plus concomitant and adjuvant temozolomide is a feasible treatment for elderly patients with newly diagnosed glioblastoma presenting with good prognostic factors.

The landmark Stupp study demonstrated a survival advantage with concomitant and adjuvant temozolomide with standard radiotherapy in GBM patient but excluded those older than 70 years. Cao et al. identified 112 patients who received hypofractionated RT, with 57 receiving concurrent and adjuvant TMZ and 55 without concurrent chemotherapy. Median overall survival among the RT+TMZ patients was 6.9 months; median OS was 9.3 months. Subgroup analysis revealed patients treated with initial hypofractionated radiation with salvage TMZ increased median OS of 13.3 months. These results suggest concurrent and adjuvant TMZ do not confer a survival benefit in elderly GBM patients. A sequential approach may be more effective and efficient strategy by selecting responding patients [20].

## 5. Chemotherapy and Supportive Care

Temozolomide (TMZ) is an oral chemotherapy with a limited side effect profile that has become the standard of care in GBM treatment. Molecular markers, such as O6-methylguanine-DNA methyltransferase methylation status, can be helpful in predicting tumor response to TMZ and therefore guides clinical decision making. In a recent phase II study the benefits of temozolomide in elderly patients (age over 70 years) seem to be associated with increased survival, especially in patients with methylated MGMT [21]. Finally, Cao et al. analyzed the efficacy and safety of RT with TMZ for elderly patients. In the study, 60 patients ≥65 years old with newly GBM were treated with RT plus TMZ. The average age was 70 (range 65–82); ECOG PS was 0-1 in 35 patients and 2 in 25 patients; complete surgery was performed in 35 patients, while partial surgery was performed in 25 patients. For all patients, PFS and overall survival OS were 9.5 and 12.7 months, respectively. OS was 13.7 and 12.4 months in patients receiving RT within 6 or more weeks from surgery, respectively. PFS was 9 versus 10 months and OS was 11.7 versus 13.7 ms, for patients treated with 40 Gy and 60 Gy, respectively. Regarding toxicity, haematological toxicity of grades 3-4 was 9% versus 23%, severe asthenia was 9% versus 15%, and nausea/vomiting was 3% versus 4% of patients receiving RT 40 Gy and 60 Gy. RT plus TMZ seems to be effective and safe in elderly patients with GBM and good ECOG PS [22].

Uzuka et al. [23] analyzed in a multicentric retrospective study the treatment outcome in elderly patients (see Table 1). The study population consisted of 79 glioblastoma patients aged >76 years. The median preoperative Karnofsky performance status score was 60. Sixty-seven patients received radiotherapy and 45 patients received chemotherapy. Patients aged >78 years old were less likely to receive radiotherapy. Patients with a postoperative KPS score >60 were significantly more likely to receive maintenance chemotherapy. The finding of this study suggests that postoperative KPS score is an important prognostic factor for glioblastoma patients aged >76, and these patients may benefit temozolomide therapy.

Yin et al. [24] demonstrated in a meta-analysis the noninferiority of TMZ alone compared to RT in improving OS, but it was not a straightforward solution for elderly GBM patients because of an increased hematological toxicities. MGMT testing might be helpful for determining individualized treatment. Two randomized clinical trials and three comparative studies were included in the analyses, which revealed an overall survival advantage for TMZ compared with RT. Most elderly patients tolerated TMZ despite an increased risk of grades 3-4 toxicities, especially hematological toxicities. In the MGMT analysis, methylated tumors were associated with longer OS than unmethylated tumors among elderly patients receiving TMZ monotherapy.

Two recent published European randomized controlled trials compared single-modality treatment in the up-front treatment of elderly patients with glioblastoma. The Nordic trial prospectively randomized patients older than 60 years [25]. In this study, 342 patients were enrolled, of whom 291 were randomised across three treatment groups (temozolomide *n* = 93, hypofractionated radiotherapy *n* = 98, and standard radiotherapy *n* = 100) and 51 of whom were randomised across only two groups (temozolomide *n* = 26 and hypofractionated radiotherapy *n* = 25). In the three-group randomisation, in comparison with standard radiotherapy, median overall survival was significantly longer with temozolomide (8.3 months, 95% CI 7.1–9.5; *n* = 93) versus 6.0 months (95% CI 5.1–6.8; *n* = 100; HR: 0.70, 95% CI 0.52–0.93, *P* = 0.01) but not with hypofractionated radiotherapy (7.5 months 95% CI 6.5–8.6; *n* = 98; HR 0.85, 0.64–1.12, *P* = 0.24). For all patients who received temozolomide or hypofractionated radiotherapy (*n* = 242) overall survival was similar (8.4 months (95% CI 7.3–9.4; *n* = 119) versus 7.4 months 95% CI 6.4–8.4; *n* = 123; *R* 0.82, 95% CI 0.63–1.06; *P* = 0.12). For age older than 70 years, survival was better with temozolomide and with hypofractionated radiotherapy than with standard radiotherapy (HR for temozolomide versus standard radiotherapy 0.35 95% CI 0.21–0.56, *P* < 0.0001); hazard ratio for hypofractionated versus standard radiotherapy was 0.59 (95% CI 0.37–0.93, *P* = 0.02). Patients treated with temozolomide who had tumour MGMT promoter methylation had significantly longer survival than those without MGMT promoter methylation (9.7 months, 95% CI 8.0–11.4) versus 6.8 months (95% CI 5.9–7.7; HR 0.56; 95% CI 0.34–0.93, *P* = 0.02), but no difference was noted between those with methylated and unmethylated MGMT promoter treated with radiotherapy (HR 0.97, 95% CI 0.69–1.38; *P* = 0.81). As expected, the most common grade 3 and grade 4 adverse events in the temozolomide group were neutropenia (*n* = 12) and thrombocytopenia (*n* = 18). Grade 3 and grade 5 infections in all randomisation groups were reported in 18 patients. Two patients had fatal infections (one in the temozolomide group and one in the standard radiotherapy group) and one in the temozolomide group with grade 2 thrombocytopenia died from complications after surgery for a gastrointestinal bleed. Standard radiotherapy was associated with poor outcomes, especially in patients older than 70 years. Both temozolomide and hypofractionated radiotherapy should be considered as standard treatment options in elderly patients with glioblastoma. MGMT promoter methylation status might be a useful predictive marker for benefit from temozolomide.

The German study NOA-08 randomized 373 patients older than 65 years with newly diagnosed anaplastic astrocytoma or glioblastoma and a performance status of 60 or greater to either radiotherapy alone or single-agent temozolomide. The authors concluded that temozolomide was noninferior to radiotherapy alone in this patients group. In the study patients were enrolled with confirmed anaplastic astrocytoma or glioblastoma, age older than 65 years, and a Karnofsky performance score of 60 or higher. Patients were randomly assigned 100 mg/m(2) temozolomide, given on days 1–7 of 1 week on, 1 week off cycles, or radiotherapy of 60 Gy, administered over 6-7 weeks in 30 fractions of 1.8–2.0 Gy. The primary endpoint was overall survival. Of 584 patients screened 412 were enrolled. Of these patients, 373 patients (195 were randomly allocated to the temozolomide group and 178 to the radiotherapy group) received at least one dose of treatment and were included in efficacy analyses. Median overall survival was 8.6 months in the temozolomide group versus 9.6 months in the radiotherapy group (*P* = 0.033). Median event-free survival did not differ significantly between the temozolomide and radiotherapy groups. Median event-free survival (EFS) did not differ significantly between the temozolomide and radiotherapy groups (3.3 months; 95% CI 3.2–4.1) versus 4.7 (95% CI 4.2–5.2; HR 1.15, 95% CI 0.92–1.43, *P* = 0.043). Tumour MGMT promoter methylation was seen in 73 (35%) of 209 patients tested. MGMT promoter methylation was associated with longer overall survival than was unmethylated status (11.9 months versus 8.2 months, *P* = 0.014). EFS was longer in patients with MGMT promoter methylation who received temozolomide than in those who underwent radiotherapy (8.4 months, 95% CI 5.5–11.7 versus 4.6, 95% CI 4.2–5.0), whereas the opposite was true for patients with no methylation of the MGMT promoter (3.3 months versus 4.6 months). From this study it emerged that temozolomide alone is noninferior to radiotherapy alone in the treatment of elderly patients with malignant astrocytoma. MGMT promoter methylation seems to be a useful biomarker for outcomes by treatment and could aid in decision-making [26].

Two studies by French ANOCEF study group in elderly patients with glioblastoma compared temozolomide alone versus best supportive care and recently, they analyzed temozolomide plus bevacizumab. In this study, patients aged 70 years or older with newly diagnosed GBM and postoperative Karnofsky performance score (KPS) less than 70 were eligible. Treatment consisted of 150 to 200 mg/m(2)/d temozolomide for 5 days every 4 weeks until disease progression. Radiotherapy was not administered. The primary end point was overall survival, and the secondary end points included progression-free survival, safety, quality of life, and cognition. Seventy patients (median age, 77 years; median KPS, 60) were enrolled. Grade 3 and grade 4 neutropenia and thrombocytopenia occurred in 13% and 14% of patients, respectively. Median PFS was 16 weeks, and median OS was 25 weeks, comparing favorably with a 12- to 16-week OS expected from a purely supportive approach. Twenty-three patients improved their KPS by 10 or more points, and 18 became capable of self-care (KPS ≥ 70). Overall quality of life and cognition improved over time before disease progression. In the 31 tumors evaluated for MGMT promoter methylation, a methylated status indicated longer PFS (26 versus 11 weeks) and OS (31 versus 19 weeks). In conclusion from this study it emerged that temozolomide has an acceptable tolerance in elderly patients with GBM and KPS less than 70. It is associated with improvement of functional status in 33% of patients and appears to increase survival compared with supportive care alone, especially in patients with methylated MGMT promoter [27].

Fotemustine (FTM) is a common treatment option for glioblastoma patients refractory to TMZ. Fotemustine is a new chloroethylnitrosourea characterized by the grafting of a phosphonoalanine group onto a nitrosourea radical. Clinical studies using fotemustine have been conducted in malignant glioma, brain metastasis of non-small cell lung cancer, and disseminated malignant melanoma. In recurrent malignant glioma, fotemustine has been used as a single agent. In the study of Santoni et al. glioblastoma patients older than 65 years were retrospectively analyzed, receiving FTM every week for 3 consecutive weeks (induction phase) and then every 3 weeks (70–100 mg/m^2^), as second-line treatment. 65 glioblastoma patients (median age, 70 years; range, 65–79 years) were eligible for this analysis. After induction, disease control rate was 43.1%. Median survival from the beginning of FTM therapy was 7.1 months, while the median progression-free survival was 4.2 months, and the 6-months progression-free survival rate was 35.4%. The most relevant grade 3 and grade 4 toxicity events were thrombocytopenia and neutropenia. In the univariate and multivariate analysis, time from radiotherapy to FTM, number of TMZ and FTM cycles, and disease control resulted in independent prognostic factors. This study showed that FTM is a valuable therapeutic option for elderly glioblastoma patients, with a safe toxicity profile [28].

Elderly patients diagnosed with high-grade gliomas have a poor prognosis and limited life expectancy and often experience rapid decline in function. Common signs and symptoms in the end of life phase of neurologic patients are raised intracranial pressure, seizures, confusion, cognitive deficits, and impaired motor function. Supportive treatment of these symptoms (such as analgesic drugs, dexamethasone, and antiepileptic and neuroleptic drugs) is of major importance to maintain quality of life as long as possible [29]. Corticosteroids and antiepileptics (AEs) are mainly used in these patients to limit tumoral edema, improve functional status, and control headaches. AEs are systematically used for a short time after cerebral surgical resection of a lesion. There is no indication for systematic use of AEs for primary prevention in patients with no history of seizures. However, in cases of seizure linked to tumoral process, use of AEs is mandatory. A recent publication has reported a potential beneficial effect of valproate acid, depending on histone deacetylase-inhibitor effect. Valproate acid use during RT for GBM was associated with improved OS, independently of RTOG recursive partitioning analysis, seizure history, and concurrent TMZ use [30].

## 6. Neurocognitive Function and Quality of Life Aspects in Elderly Patients

In addition to the neurological complication related to brain tumors themselves, the treatments are often associated with harmful effects on the central nervous system that can lead to cognitive impairments. These patients may experience both acute and late toxicities, resulting both from direct toxic effects on the nervous system and indirect dysfunction (e.g., metabolic dysregulation and cerebrovascular disorders). Toxicity from brain tumor therapy may range from focal neurological deficits to generalized neurological syndromes. These frequently include fatigue and cognitive impairment [31]. A baseline impaired cognitive function is common among older patients presenting for medical treatment. Cognitive function has a significant practical implication for older patients receiving treatment for cancer. In the presence of memory impairment, the patient will have difficulty in understanding and remembering treatment instructions, potentially affecting compliance with oral cancer therapy or supportive medication. Patients with cognitive impairment may have difficulty in remembering the signs and symptoms of cancer therapy side effects that warrant medical attention or may have trouble in remembering appointments increasing caregiver burden. Cognitively impaired patients receive less definitive cancer care than others. Cognitive disorders in older patients present prior to cancer treatment are often underdiagnosed without screening.

In many studies, the quality of life (QOL) in brain tumor patients is assessed with Karnofsky performance status score, although more specific tools are now available, such as EORTC QLQ C30 and the EORTC brain cancer module (EORTC QLQ-BN20). The neurocognitive function is usually assessed through the Folstein Mini-Mental Status Examination (MMSE), but this may underestimate the proportion of patients with actual cognitive decline [32].

Many factors contribute to neurocognitive outcome, such as direct and indirect tumor effects, seizures, medications, and oncological treatment. Although the role of high-dose radiotherapy on neurological impairment is well established, the impact of other treatments is less clear. There is increasing interest in the measure of neurocognitive outcomes in brain tumor patients, due to the strong relationship between neurocognitive status and health-related quality of life.

Recently, Minniti et al. [33] assessed the health-related quality of life (HRQOL) in elderly patients with GBM treated with short-term RT with concomitant temozolomide, and HRQOL was assessed by European Organisation for Research and Treatment of Cancer (EORTC) Quality of Life Core-30 (QLQ-C30, version 3) and EORTC Quality of life questionnaire Brain Cancer Module (QLQ-BN20). 9 domains were preselected (global QLQ, social functioning, cognitive functioning, emotional functioning, physical functioning, motor disfunction, communication deficit, fatigue and insomnia) and changes in these domains have been evaluated by various assessment (4 weeks after RT and thereafter every 8 weeks during the treatment until disease progression). A change of ≥10 points (on a scale of 0 to 100) was classified as the minimum clinically meaningful change in the mean value of a HRQOL parameter. Sixty-five patients completed the questionnaires at baseline showing that physical functioning and cognitive functioning were the most impaired deficits at baseline. Results of statistical analysis show an improvement of important HRQOL domains until the time of disease progression. The improvement in QOL and cognition before disease progression during treatment with TMZ in elderly patients was also revealed in a French study.

In this study, patients aged 70 years or older with newly diagnosed GBM and postoperative KPS less than 70 were eligible for treatment only with temozolomide for 5 days every 4 weeks until disease progression. Qol and cognition were evaluated with EORTC QLQ-C30 and BN20 and MMSE. Results show that the KPS increased over time during treatment period. In absolute values KPS improved in 23 of 70 patients (32,9%) with a median improvement of 20 points. The median duration of such improvement was 4 months. Eighteen patients achieved a KPS ≥ 70, indicating that they were capable of self-care. This improvement lasted for a median of 3 months. The patients cognitive function improved over time, on the basis of MMSE evaluation, (*P* < 0.001). For QOL questionnaires the baseline compliance rate was 84%, but this rate declined with time. Scores related to global QOL improved significantly over time (*P* = 0.01), as did physical (*P* = 0.003), role (*P* = 0.02), cognitive (*P* = 0.006), and social (*P* < 0.001) functioning scores. No significant changes were observed in the other domains. Regarding the QLQ-BN20 questionnaire, the scores on motor dysfunction (*P* = 0.006), drowsiness (*P* = 0.001), and bladder control (*P* = 0.03) also improved over time before disease progression.

In the NOA-08 trial elderly patients with anaplastic astrocytoma or GBM were randomised to receive dose-dense TMZ or standard RT [26], owing no significant changes between groups in the HRQOL analysis in terms of emotional and social function and global QOL, while patient treated with chemotherapy reported more physical adverse events such as fatigue and nausea.

At our Institute, Istituto Oncologico Veneto, Padua, a periodical evaluation of QOL, cognitive function and emotional status of elderly patients with brain tumor, has become a standard practice. Particularly, we perform a baseline evaluation, followed by new examination at each radiological evaluation. The geriatric assessment, made at baseline, includes the classical Comprehensive Geriatric Assessment, as defined by Monfardini et al. [34] and the EORTC-QLQ C30 and Brain Cancer Module EORTC-QLQ BN20 [35].

The evaluation of elderly patient is done through the use of the comprehensive geriatric assessment scale (CGA), developed and validated by the Italian Group for Geriatric Oncology (GIOGer) [36], an instrument that assessed demographic characteristics, physical performance, disability indexes, and depression and cognitive status, helping for the identification of elderly patients suitable for standard treatment (fit patients) and those suitable only for supportive care (frail patients) [37]. A great number of elderly patients with cancer fall however into the category of “vulnerable” patients with different degrees of functional impairment that can influence morbidity and mortality and for whom an individualized approach must be prosecuted in order to provide optimal treatment. We prospectively collect data on this complete assessment to explore the impact of QOL and cognitive function as prognostic factors in brain tumors.

## 7. Conclusion

Treatment for elderly patients with primary brain tumors should be individualized and age alone should not preclude the use of more aggressive treatments. In the elderly patients, no standard treatment has been established and therefore there are actually many studies to evaluate treatment regimes and outcomes in elderly glioblastoma patients. The best treatments could be efficiently proposed according to different factors as comorbidities or molecular factors. Patients with little comorbidity could be treated aggressively, even if their performance status is poor. However frail patients with previous loss autonomy linked to severe comorbidities should be recommend less aggressive treatments. Therefore, in the elderly patients a geriatric evaluation can help to define elderly patients more accurately than the performance status. Supportive care plays an important role in the management of glioma in elderly patients.

## Figures and Tables

**Table 1:** Data on outcome in elderly patients affected by glioblastoma.

Author	Therapy	PTS	KPS	Median age (years)	PFS (ms)	OS (ms)
Uzuka et al. [23]	RT or CT	79	60	78	6.8	9.8
Minniti et al. [19]	RT (60 Gy) + TMZ	32	80	74	7	10.6
Malmstrom et al. [25]	RT (34 Gy) versus RT (60 Gy) versus TMZ	342	60–100	70	NA	7.5 versus 6 versus 8.3
Lombardi et al. [38]	RT (60 Gy) + TMZ versus TMZ alone	60	80	70	12.7 versus 9.5	12.4 versus 13.7
Wick et al. [26]	RT versus TMZ alone	584	60	65	3.3 versus 4.7	8.6 versus 9.6

PFS: progression-free survival; OS: overall survival; KPS: Karnofsky Performance Status; RT: radiotherapy; TMZ: temozolomide; NA: not available.
